# Early diagnosis and response assessment in chronic recurrent multifocal osteomyelitis: changes in lesion volume and signal intensity assessed by whole-body MRI

**DOI:** 10.1259/bjr.20211091

**Published:** 2021-12-16

**Authors:** Angelina Kieninger, Jürgen F. Schäfer, Ilias Tsiflikas, Monika Moll, Jasmin Kümmerle-Deschner, Mareen S. Kraus, Michael Esser

**Affiliations:** 1 Department of Diagnostic and Interventional Radiology, University Hospital Tübingen, Tübingen, Germany; 2 Department of Pediatrics, Filderklinik, Filderstadt-Bonlanden, Germany; 3 Department of Pediatrics, University Hospital Tübingen, Tübingen, Germany

## Abstract

**Objective:**

To assess the effectiveness of whole-body MRI (WB-MRI) in early diagnosis of chronic recurrent multifocal osteomyelitis (CRMO) and the prediction of clinical response through quantitative MRI features.

**Methods:**

20 children (mean age, 10.3 years; range, 5–14 years) with CRMO underwent WB-MRI and were assessed with a clinical score (Jansson) at baseline (median time after first encounter, 8 months) and follow-up (median time after baseline, 11.5 months). Baseline WB-MRI scans were classified as early (within 6 months after first encounter) and late. Clinical responders and non-responders were compared regarding number and localization of bone lesions, lesion volume and T2 signal intensity (SI) ratio (lesion to muscle).

**Results:**

Diagnosis of CRMO was made promptly in the early WB-MRI group (*n* = 10; median, 3 months) compared to the late WB-MRI group (*n* = 10; 18 months; *p* = 0.006). Bone lesions were mainly located in the lower extremities (*n* = 119/223; 53%). No significant difference was detected regarding the number of bone lesions and lesion volume in the subgroups of clinical responders (*n* = 10) and non-responders (*n* = 10). Responders showed a higher volume reduction of bone lesions at follow-up compared to non-responders (*p* = 0.03). Baseline and follow-up SI ratios were lower in responders (5.6 and 5.8 *vs* 6.1 and 7.2; *p* = 0.047 and *p* = 0.005).

**Conclusion:**

The use of WB-MRI within 6 months of disease suspicion may serve as a benchmark to support early diagnosis of CRMO. T2 SI ratios and the reduction of lesions’ volume correlate with clinical outcome.

**Advances in knowledge:**

WB-MRI at an early stage of suspected CRMO plays a key role for early diagnosis. This is the first study showing that quantitative MRI features are suitable for response assessment and can be used as prognostic markers for the prediction of clinical response.

## Introduction

Chronic recurrent multifocal osteomyelitis (CRMO), also called chronic non-bacterial osteomyelitis (CNO), is a rare, aseptic, auto-inflammatory bone disease affecting infants and adolescents. Patients often present with non-specific symptoms such as episodes of localized pain, soft tissue swelling and restricted range of movement, which makes the diagnosis challenging. Depending on the time of diagnosis, these periods may last from days to several years.^1^ An early diagnosis is not only important for treatment, but may also prevent complications and strongly impacts the long-term outcome of the disease.^2^ Histopathological findings and serum biomarkers are also not specific for the diagnosis of CRMO. To date, the diagnosis of CRMO is often made clinically and radiologically excluding other diseases, such as infections and neoplasia.^3^ In some cases, bone biopsy cannot be avoided; however, it should not be used as a routine diagnostic tool when CRMO is considered.^4,5^ Whole body imaging is indicated as an alternative to detect symptomatic as well as clinically silent (occult) multifocal bone lesions.^6–8^ In the literature, whole-body MRI (WB-MRI) was considered to be superior to radiography and bone scintigraphy revealing characteristic patterns of bone involvement, while targeted imaging was shown to underestimate the extent and severity of the disease.^4–6,9^ WB-MRI has a high sensitivity for small soft-tissue lesions and bone marrow edema; it detects the typical bilateral symmetric distribution of CRMO lesions and can illustrate associated findings like spinal involvement, synovitis and sacroiliitis.^5,6^ Further advantages of WB-MRI include technical aspects (non-invasive, radiation-free) and therapy response monitoring.^4,10^

When MRI was first introduced for CRMO patients, the modality was considered useful not only in assessing the extent and activity of CRMO, but also aiding disease monitoring.^11^ Today, WB-MRI has become essential and the most important diagnostic tool in the management of CRMO in children.^7,12^ Nevertheless, CRMO is often underrepresented in the differential diagnosis of pediatric bone pain.^2^ A late diagnosis with an increased risk for complications and late-effects is still an issue of concern.^13,14^ To date, there is no consensus or recommendation on the ideal time to perform a WB-MRI during the diagnostic workup of bone pain in children. Another open question refers to whether WB-MRI can be used as a prognostic tool in CRMO patients.^12^ While a few studies attempted to correlate imaging patterns and lesion locations with clinical factors, to the best of our knowledge WB-MRI has not been evaluated as a quantitative tool for the prediction of therapy response. We hypothesized, firstly, that WB-MRI – within 6 months after the first encounter – is favorable for an early diagnosis of CRMO and, secondly, that volume and intensity changes of bone lesions are a possible prognostic marker.

Therefore, the aim of this study was to assess the effectiveness of WB-MRI in early diagnosis of CRMO and the prediction of clinical response through quantitative MRI features.

## Methods and materials

This retrospective study was approved by the local ethics committee (project number 694/2011A). All patients or legal guardians gave signed consent for MRI, however, informed consent for the study participation was waived by the ethics committee. All study procedures were conducted in accordance with the ethical standards as laid down in the Declaration of Helsinki in 1964 and its later amendments.

### Patient selection

A retrospective patient cohort was selected from our radiology information system between January 2005 and December 2013. Inclusion criteria were age under 18 years, diagnosed CRMO according to published criteria^3,15^ and at least two WB-MRI examinations. This led to a cohort of 20 patients (mean age, 10.3 years; range, 5–14 years; interquartile range, IQR, 3 months; 16 girls, 4 boys). Patient characteristics are displayed in Table 1.

**Table 1.** Patient characteristics (sorted by number of CALs)

Patient no.	Sex	Age (y)^a^	CAL (n)^b^	Symptomatic sites	Medication	WB-MRI lesions (n)^b^	Diagnosis group	Encounter to baseline (mo)	Response group
**1**	F	12	14/0	Sternum, sacroiliac joint, hip, ankle, metatarsal bones	Ibuprofen/Naproxen, Sulfasalazine	40/24	Early	3	R
**2**	F	10	11/1	Sacroiliac joint, ankle	Naproxen	22/10	Late	9	NR
**3**	M	8	7/3	Distal femur, ankle, ribs	Ibuprofen	14/10	Late	44	NR
**4**	F	9	5/2	Thoracic spine	Naproxen/Ibuprofen, Sulfasalazine/Methotrexate	17/0	Late	23	NR
**5**	F	11	5/5	Thoracolumbar spine	Naproxen	10/2	Early	1	NR
**6**	F	9	5/0	Mandibula	Naproxen, Methotrexate, Bisphosphonate	5/0	Late	24	R
**7**	F	10	4/0	Humerus, sacrum	Naproxen	15/15	Late	25	R
**8**	F	13	4/0	Pelvis	Clindamycin	8/3	Early	6	R
**9**	M	12	4/2	Proximal tibia, knee, calcaneus	Naproxen	8/6	Early	1	NR
**10**	F	11	3/0	Pelvis, ankle	Naproxen	6/5	Early	2	R
**11**	F	14	3/0	Sternum, sacroiliac joint, hip	Naproxen, Prednisolone, Sulfasalazine	3/0	Early	1	R
**12**	F	12	3/1	Sacroiliac joint	Ibuprofen, Sulfasalazine	9/9	Early	6	NR
**13**	F	9	3/3	Hip, ankle, calcaneus	Naproxen	3/1	Late	30	NR
**14**	F	9	3/2	Sternum, hip	Ibuprofen	6/8	Early	5	NR
**15**	F	8	2/0	Ankle	Ibuprofen, Bisphosphonate	15/13	Early	3	R
**16**	M	12	2/3	Sacroiliac joint	Naproxen	2/3	Early	4	NR
**17**	F	5	1/0	Distal femur	Naproxen	6/6	Late	13	R
**18**	F	11	1/1	Clavicle	Naproxen	10/1	Late	1	NR
**19**	F	9	0/0	None	Naproxen, Sulfasalazine	4/2	Late	9	R
**20**	M	12	0/0	None	Naproxen, Sulfasalazine	3/2	Late	3	R

CAL, clinically active lesion; F, female; M, male; NR, clinical non-responder; R, clinical responder; WB-MRI, whole-body MRI.

a At time of the onset

b Baseline/Follow-up

**Figure 1.**
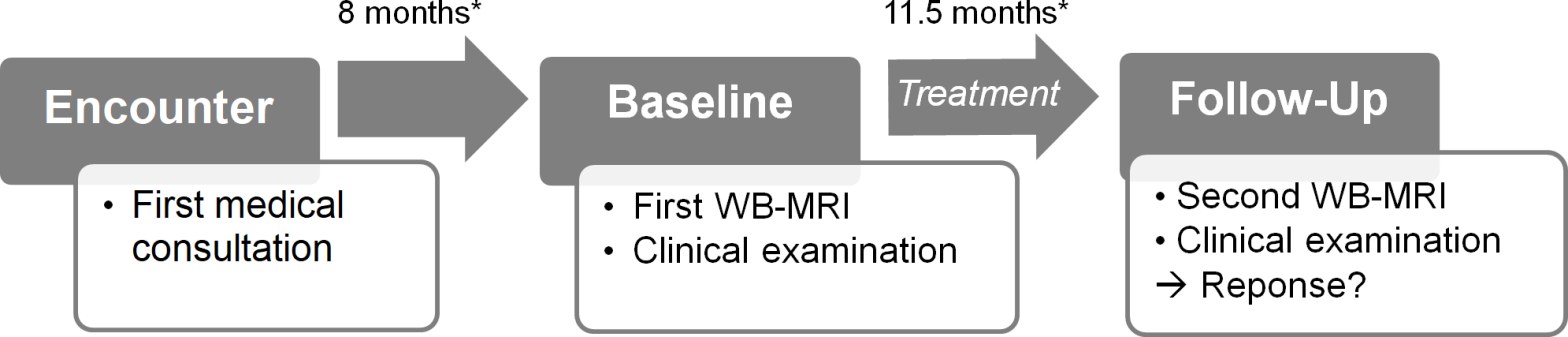
A–B Timeline of procedures in the study. Time of first symptom occurrence was noted as the clinical onset of disease. Patients were assessed clinically and by imaging at the time point of the first and follow-up WB-MRI. Patients were under medication between baseline and follow-up. Clinical response evaluation was performed at follow-up. Median periods of time (*) between onset and baseline as well as between baseline and follow-up are given. WB-MRI, whole-body MRI.

Patients were assessed clinically and by imaging at the time point of the first WB-MRI (baseline; median time after first encounter, 8 months; IQR, 23.8 months) and the second WB-MRI (follow-up; median time after baseline, 11.5 months; IQR, 12.3 months). Procedures are illustrated on a timeline in Figure 1. Imaging was performed as part of the diagnostic work-up of the clinical partner. 19 of 20 patients were under medication following Jansson et al^16^ between baseline and follow-up. One patient was treated with antibiotics only due to primarily suspected bacterial osteomyelitis (Table 1).

### Clinical examination

Patients were assessed by two pediatricians with 5 (MM) and 10 (JK) years of experience in pediatric rheumatology. Physical examination and laboratory tests were performed as part of the composite score published by Jansson et al.^3,15^ Clinical active lesions (CALs) were defined as anatomical regions with local pain, swelling, overheating or limited range of motion. The time of the first medical consultation was considered as the first encounter and the time point of CRMO diagnosis according to the Jansson criteria was also recorded. Clinical responders were defined as patients who had no more symptoms in the follow-up evaluation. All other patients - with at least one CAL - were defined as clinical non-responders.

### WB-MRI image acquisition

All WB-MRI examinations were performed on a 1.5T scanner (Magnetom Avanto; Siemens Healthineers, Erlangen, Germany) using the manufacturer’s head and body array coil with automatic coil selection. Patients were positioned in supine position with arms placed along the chest and with the hands on the lower abdomen. Both feet were positioned in a lateral view for the coronal short tau (inversion time) inversion-recovery (STIR) sequence.^12^

All MRI examinations included a coronal whole-body STIR as the standard sequence performed in five stacks (head/neck, thoracic, abdominal, pelvic, and upper and lower leg region). For a better imaging of osteitis, periosteal reaction and extraosseous findings, contrast enhanced 3D *T*_1_ weighted Dixon–volume-interpolated breath-hold examination (Dixon-VIBE) sequences were performed in axial orientation for the whole body in five to six stacks (head/neck, thoracic, abdominal, pelvic, upper and lower leg region) following the S1 Guideline for WB-MRI in children and adolescents.^17^ Imaging parameters are summarized in Table 2. Contrast media was adapted to the body-weight accordingly (0.1 mmol/kg; gadobutrol; Bayer Healthcare, Berlin, Germany) and was injected intravenously using an automated injector pump (Medrad, Spectris Solaris; EP MR Injector System; Bayer Healthcare, Berlin, Germany). Total acquisition time ranged between 30 and 50 min.

**Table 2.** Sequence parameters of whole-body MRI protocol

Variables	2D STIR	*T*_1_W Dixon-VIBE CE
**Plane**	coronal	axial
**Repetition time, ms**	7330	6.6
**Inversion time, ms**	180	N/A
**Echo time, ms**	85	2.3
**Echo train length**	19	1
**Flip angle, degrees**	150	10
**Image matrix, pixels**	384 × 269	288 × 192
**Pixel spacing, mm**	1.25 × 1.25	1.5 × 1.5
**Slice thickness, mm**	3	2

CE: contrast-enhanced;STIR: short tau inversion-recovery; VIBE: volume-interpolated breath-hold examination.

### Image analysis

Images were reviewed on a standard PACS-workstation by a pediatric radiologist with over 20 years of experience in pediatric imaging (JFS) and a post-graduate physician assistant (AK). The unexperienced reader was previously trained for the lesion measurements as described below. Discrepancies were solved by the consensus reading. The readers were blinded to all identifying data, the group type (early *vs* late) and results of the clinical examination.

Baseline MRI examinations were classified in relation to the time point of the patient´s first encounter: primary WB-MRI examinations that were performed within 6 months after the first presentation were classified as “early WB-MRI”, other baseline examinations were allocated to the “late WB-MRI” group.

**Figure 2.**
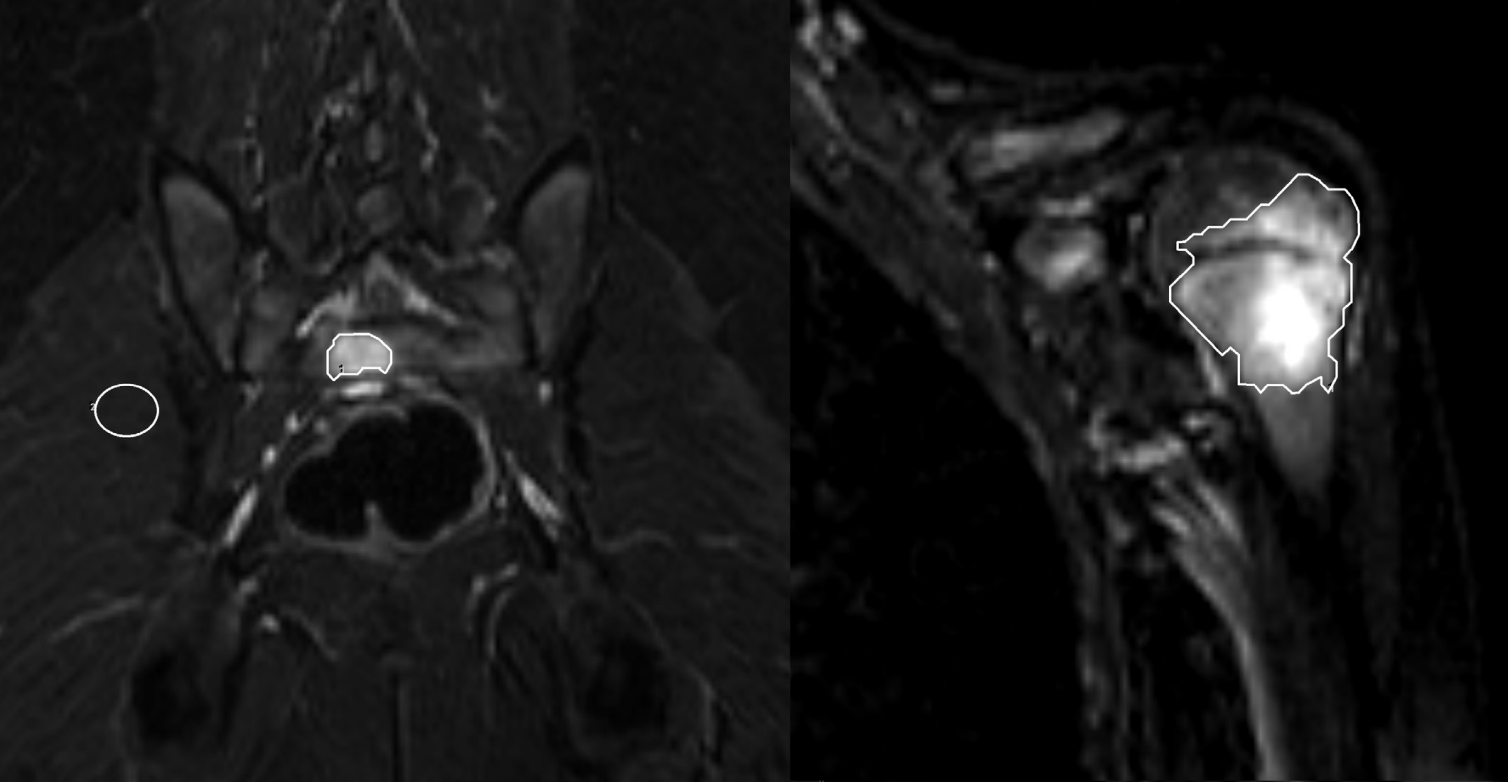
A–B A 10-year-old girl with local pain of the left humerus and the pelvis. Representative single slices with polygonal ROIs of the coronal T2 STIR images comprising the complete hyperintense lesions of the (**A**) sacrum and (**B**) the left proximal humerus with epimetaphyseal location. On the left side (**A**) the corresponding signal intensity measurement of the adjacent muscle is displayed for the calculation of the signal intensity ratios. Signal intensity values were 5.4 at the sacrum (**A**) and 5.1 in the left humerus. Additional bone lesions were found in the left clavicle, the right proximal humerus, the femoral bones and the both tibiae. ROI, region of interest; STIR, short tau inversion-recovery.

Bone lesions were deﬁned as focal areas of abnormal bone marrow signal intensities, such as hyperintensity on coronal STIR images (edema-like) and areas of contrast enhancement. The number of lesions per patient and the localization of each lesion were classified according to the affected area (head, extremity, trunk, spine), the side (right, left) and the bone with geographical region (epiphyseal, metaphyseal, diapyhseal, apophyseal). Bone deformities and fractures were recorded as complications. Symmetry of lesions was deﬁned as a bilateral involvement of periarticular areas. The signal intensity (SI) ratios were calculated as the average of the lesions T2 SI divided by the average SI of the adjacent muscle measured on coronal STIR images. Hence, polygonal regions of interest (ROIs) of the lesions were drawn on each imaging slice to cover the lesion entirely (Figure 2). The boarder of a lesion was determined as accurately as possible using the interruption of regular T2 bone marrow signal as a border line and there defining the beginning of the lesion. Afterwards, the geometrical mean SI value of all slices was calculated to minimize the statistical influence of scattering. To determine the three-dimensional volume, the surface areas of all ROIs (mm^2^) were multiplied with the slice thickness. Total volume and signal intensity were determined only in lesions with at least 10 mm^2^ diameters at baseline and follow-up MRI.

### Statistical analysis

For statistical analysis, SAS jmp (v. 13 for Mac Os X, SAS Institute Inc., Cary, NC) was used. The Shapiro–Wilk test was used to assess the distribution of quantification data. Continuous variables are presented as means and range. Data that did not follow a normal distribution are presented as median with IQRs.

The *t*-test was used for paired samples with normal distribution, the sign test and Wilcoxon-sign-test for paired samples without normal distribution. Wilcoxon–Mann–Whitney-test was performed for unpaired samples.

A *p*-value less than 0.05 was considered to indicate statistical significance.

## Results

### Early *vs* late WB-MRI

10 patients (50%) received an early baseline WB-MRI (within 6 months after first encounter; median 2.5 months; IQR, 3.3 months; eight girls, two boys; mean age, 11.4 years). For the other 10 patients, baseline WB-MRI was performed later than 6 months after first encounter (median 26 months; IQR, 28.8 months; eight girls, two boys; mean age, 9.2 years). For further detail, see Table 1.

On average, diagnosis was made 10.7 months after first encounter (median, 5.5 months; range, 1–44 months). In the subgroup of patients with an early WB-MRI, diagnosis of CRMO was made within 3 months (median; IQR, 4.3 months), in the late WB-MRI group diagnosis was established 18 months after first encounter (IQR, 18.8 months; *p* = 0.006). In all patients with early WB-MRI (*n* = 10), diagnosis was set within 6 months, but only two (20%) of the patients with late baseline WB-MRI were diagnosed on a clinical basis within the same time. In these cases, diagnosis was made by clinical findings, prior local MRI and bone biopsy.

### Clinically active lesions

Overall, 80 CALs were detected in the baseline clinical assessments of 18 patients (median number of lesions per patient 3; see Table 3). Most common symptoms displayed by the CALs were pain (*n* = 74/80) and local swelling (*n* = 29/80). Two patients (Table 1, Patient No. 19 and 20) did not show any symptoms at the time of the first WB-MRI and were already under medication with naproxen and sulfasalazine; however, they had presented with symptoms at the time of the clinical onset and first encounter before.

**Table 3.** Differences between clinical responders and non-responders

Variables	All patients	Responders	Non-responders	*p*-value (R *vs* NR)
**Number (n)**	20	10	10	
**Clinically active lesions (n)^a^**	80	36	44	0.34
**Clinically active lesions (n)^b^**	23	0	23	<0.0001
**WB-MRI lesions (n)^a^**	206	105	101	0.54
**WB-MRI lesions (n)^b^**	121	71	50	0.76
**Lesion volume per patient (median; ml)^a^**	28.4	29.8	27.0	0.60
**Lesion volume per patient (median; ml)^b^**	12.5	11.9	18.8	0.43
**Lesion volume difference a–b per patient (ml)**	−15.9	−17.9	−8.2	0.03
**SI ratio (median; range)^a^**	5.9 (2.2–25.5)	5.6 (2.2–14.5)	6.1 (3.1–25.5)	0.047
**SI ratio (median; range)^b^**	6.5 (2.8–20.5)	5.8 (2.9–20.5)	7.2 (2.8–16.8)	0.005
**SI ratio difference a-b**	0.6	0.2	1.1	0.82

NR: clinical non-responder;R: clinical responder; SI: signal intensity; WB-MRI: whole-body MRI.

a Baseline.

b Follow-up.

When having a closer look at response assessment, 23 CALs were detected in 10 patients (considered as clinical non-responders; mean age 10.3 years; range 8–12 years; median number of lesions per patient 2) accounting for a decrease of 71% (*p* = 0.001) compared to all lesions at the baseline setting. The most frequent sign of inflammation was localized pain (*n* = 17/23). 10 patients did not show any CALs at the follow-up (clinical responders; mean age 10.3 years; range 5–14 years).

### WB-MRI findings

**Figure 3.**
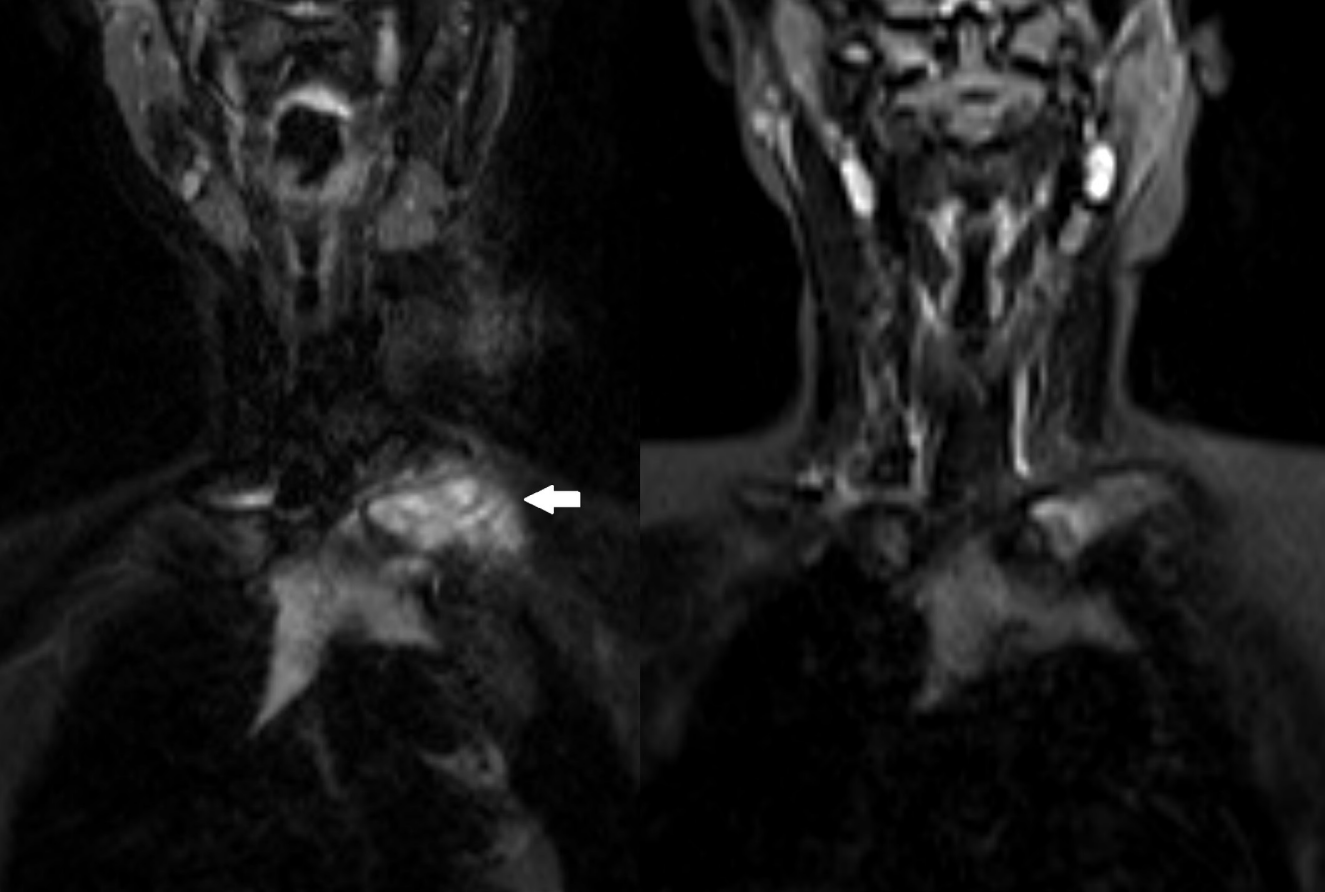
A–B Baseline and follow-up WB-MRI in a 12-year-old boy under Naproxen treatment. (**A**) Coronal *T*_2_W STIR illustrates a hyperintense bone lesion in the left clavicle (arrow) with surrounding soft tissue swelling. (**B**) Follow-up after 7 months shows a considerable reduction of T2 signal in the left clavicle and beginning normalization of the bone marrow signal under therapy. STIR, short tau inversion-recovery; WB-MRI, whole-body MRI.

In total, baseline WB-MRI detected 206 bone lesions (median number of lesions per patient, 8; range, 2–40; see Table 3). All lesions were hyperintense on T2 STIR images with an ill-defined edema-like appearance (Figures 2 and 3). No additional lesions were detected in the contrast-enhanced sequences. All patients had multifocal bone lesions. 13 patients (65%) showed at least one site with a bilateral symmetric distribution of lesions.

In the follow-up WB-MRI examination, 121 lesions were detected, which represents a decrease of 41% (*p* = 0.002; median number of lesions, 4; range, 0–24). 77 of 206 lesions in WB-MRI were detected clinically at baseline assessment (37%); in contrast, only 19 of 121 bone lesions were clinically notable at the follow-up examination (16%).

### Correlation of CALs and lesions detected on WB-MRI

In 80% of the patients (*n* = 16), more lesions were detected by WB-MRI than by clinical examination. 77 of 80 (96%) CALs were detected by WB-MRI at baseline and 19 of 23 (83%) CALs were found at follow-up WB-MRI. 9 of 10 patients without CALs at the follow-up still had bone lesions in the WB-MRI.

### Anatomical and geographical distribution

Most lesions were located in the lower extremities (*n* = 119, 53%) with the pelvis (*n* = 34; 15%) being the most frequently affected bone, followed by the proximal tibia (*n* = 29; 13%) and spine (*n* = 28, 13%). Involvement of the clavicula was found in three cases (Figure 3). Seven patients were diagnosed with scoliosis and/or thoracic hyperkyphosis. Further complications were vertebral body fractures that were found in four patients, a fractured pubic bone in one patient and a deformity of the temporomandibular joint in another case. According to the geographical classification, most lesions of the long bones appeared to be in the metaphysis (*n* = 56) and epiphysis (*n* = 48). Apophyseal lesions were found in eight cases and diaphyseal involvement in seven cases.

### Volume and signal intensity ratios

**Figure 4.**
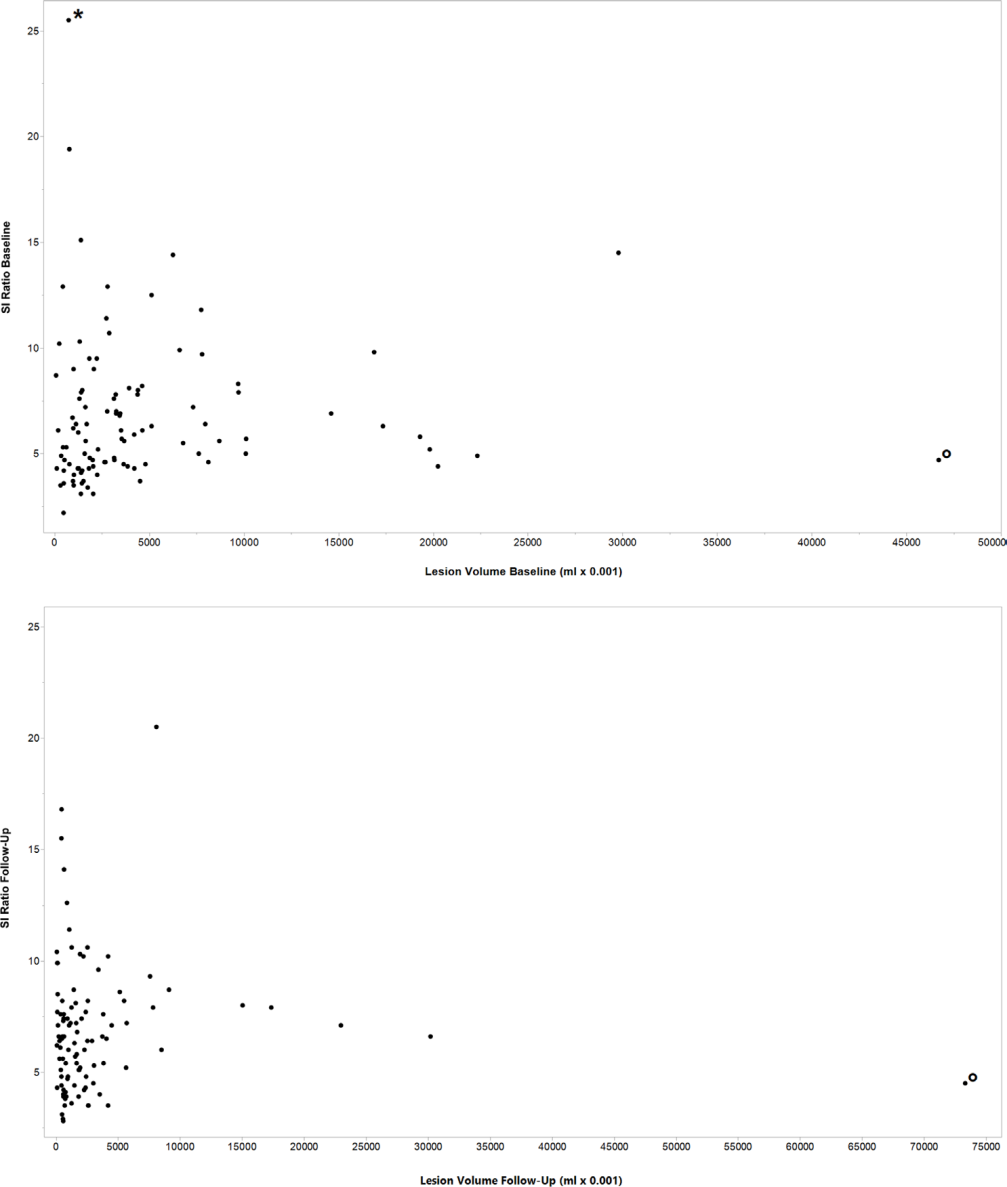
A–B Distribution of SIs and lesion volume values. Scatter plot showing the distribution of the single SI ratios and lesion volume values in the baseline (**A**) and the follow-up WB-MRI (**B**). Two lesions presented with unusual values in the data set and were marked with an asterisk and circles, one with a high SI value in the baseline (*), the other one with high lesion volume in baseline and follow-up WB-MRI (°). The latter was an extended lesion with sclerotized parts and bone edema affecting nearly the whole sacrum. SI, signal intensity; WB-MRI, whole-body MRI.

Median volume of all lesions was 28.4 ml (IQR, 32.1 ml) at baseline and 12.5 ml at follow-up (IQR, 18.8 ml) representing a median volume reduction of 44% per patient (*p* = 0.01). In the baseline setting, median SI ratio of all lesions was 5.9 (IQR, 3.5) compared to a median SI ratio of 6.5 (IQR, 3.1) in the follow-up examinations (see Table 3). To demonstrate the distribution of lesion volume and SI ratios all single values are included in Figure 4.

### Comparison of clinical responders and non-responders

Between the subgroups of clinical responders and non-responders, there was no significant difference found according to the number of bone lesions in WB-MRI (see Table 3). Two patients had an increase of detected bone lesions in WB-MRI and were clinically classified as non-responders.

There was no significant difference between clinical responders and non-responders concerning the median volume of bone lesions in WB-MRI per patient (see Table 3). The change of the median lesion volume between baseline and follow-up was different for responders compared to non-responders (*p* = 0.03) with a higher reduction of volume in the responders subgroup.

Differences in median SI ratios were found between clinical responders and non-responders at both time points - baseline and follow-up - with overall lower SI ratios in the subgroup of responders (*p* = 0.047 and *p* = 0.005; see Table 3).

## Discussion

The results of this study show that WB-MRI plays a key role in early diagnosis of CRMO by identifying typical symmetric patterns of multifocal bone lesions. For the first time, this study introduces quantitative imaging features of bone lesions in CRMO, which can be useful for the prediction of clinical response.

The time of diagnosis was on average six times longer in the late WB-MRI group compared to patients who underwent the first WB-MRI within 6 months after their first encounter. In all early WB-MRI patients, diagnosis of CRMO could be made within 6 months.

The importance of an early diagnosis for clinical response has been discussed previously.^2^ Especially for patients with spinal involvement, the start of an adequate treatment is essential to avoid complications and long-term effects. In addition, there is evidence that the response to non-steroidal anti-inflammatory drugs (NSAIDs) is influenced by the duration of active disease.^18^ In literature, there is a wide time range between clinical onset and diagnosis of CRMO, which is in line with the results of this study (1–44 months). Previous studies have reported a duration ranging from 1 month to 5 years^13^ or even up to 7 years between first symptoms and diagnosis.^14^ This indicates that CRMO is still not commonly recognized. As indicated by a survey in 2017, the relevance of WB-MRI is also underestimated among pediatric rheumatologists.^19^ Only 36% of rheumatologists “often or always” used WB-MRI when suspecting CRMO. Our results suggest to use a period of 6 months after the first encounter as a benchmark for the performance of a WB-MRI in order to ensure an early diagnosis of CRMO.

Our data underline the importance of WB-MRI to detect clinically occult lesions. As shown by previous studies,^6^ nearly all CALs had a morphological correlate in the WB-MRI (96% in baseline), but not vice versa (37%). In accordance with our results, Fritz et al found that only 33% of radiologically detected lesions were also clinically apparent.^5^ Follow-up WB-MRI in the presented work still detected bone lesions in 9 of 10 clinical responders. Our study results confirm the importance of WB-MRI for the assessment of disease activity for primary diagnosis as well as response monitoring.

Noteworthy, three CALs (4%) had no correlate in the baseline MRI. This may be explained by an amplified musculoskeletal pain syndrome, when patients develop an abnormal pain; hereby, the differentiation between pain amplification and disease activity is difficult.^7^ Interestingly, in our study, the three CALs without MRI correlates were located in the foot and ankle. This region is sometimes difficult to assess accurately, because focal T2 changes may be interpreted as remnants of red bone marrow in growing patients.^20^ Another interesting point is that all bone lesions could be detected on STIR images but no additional lesion was found in the contrast-enhanced sequences. Fritz et al already proposed that contrast agent should only be used in the baseline MRI for the assessment of organs and extraosseous findings.^5^ Our results support that contrast agent is not essential for bone lesion detection or for making the diagnosis of CRMO; however, it may be helpful when further differential diagnoses have to be excluded or the primary diagnosis remains unclear after the STIR sequence.

This is the first study to evaluate quantitative MRI parameters such as volume and SI of bone lesions in CRMO to improve lesion characterization for an early identification of clinical response. In addition, the number of bone lesions in WB-MRI alone is not a reliable marker for the clinical outcome. The decline of the WB-MRI lesion volume between baseline and follow-up was higher among clinical responders compared to non-responders (*p* = 0.03; Table 3). Accordingly, lesion volumes in the follow-up showed a trend towards lower values in the subgroup of responders (11.9 *vs* 18.8). Lower signal intensities of bone lesions and a higher reduction of the lesions’ volume in patients with clinical response indicate a correlation between quantitative imaging features and clinical outcome. These findings lead to the conclusion that lesions with a lower disease activity show a lower SI ratio which, in turn, goes along with a more favorable prognosis. The measurement of SIs at the time of the baseline may aid to predict clinical response (Table 3). In our small patient cohort, there was no relevant change of SI ratios between baseline and follow-up with a wide range of values from 2.2 to 25.5 (Figure 4). A possible explanation may be that the decline in SI occurs with a delay during the course of the disease. Our report provides a proof of concept, the analysis of a larger patient cohort or the preselection of certain lesions for measurement could validate our results enabling the use of SI as a biomarker.

Arnoldi et al have previously described a correlation between clinical appearance of CRMO and findings in WB-MRI, therefore suggesting the use of a radiologic WB-MRI index including the number and maximum size of lesions, periosteal reaction and spinal involvement.^8^ Recently Zhao et al have proposed a consensus-based MRI scoring comprising different image-based variables such as hyperintensity within bone marrow and the surrounding tissue.^21^ However, T2 hyperintensity was graded only as present or absent, but no quantification was applied. Our study further encloses two quantitative MRI parameters (volume changes and SI ratio) to be considered for a standardized radiological evaluation of CRMO. The integration in future alternative scoring systems may improve the assessment of disease activity and allow for prognosis estimation.^12^ This way of classifying response might enable a more focused and individualized treatment management in the future.^8^ One can imagine that future artificial intelligence systems with automated lesion detection may be supported by the use of SI for decision-making. In this context, normal SI ratios of disease-free persons should be obtained as reference standard to aid a reliable differentiation between bone lesions typical for CRMO and normal bone marrow in children.^12^

The following limitations need to be considered in this study: the main limitation is the small patient cohort of the study and the subgroups (responders/non-responders, early/late diagnosis), which hampers statistic evaluation and limits the generalizability of the results. Another limitation is the retrospective design of the study and the fact that image analysis was conducted by a single radiologist; therefore, inter-rater reliability was not calculated. A possible bias might arise from the baseline assessment not exactly matching the time of clinical onset or first encounter.

## Conclusion

The use of WB-MRI within 6 months of disease suspicion may serve as a benchmark to support early diagnosis of CRMO. Quantitative MRI features such as T2 SI ratios correlate with clinical outcome. They are suitable for response assessment and can be used as prognostic markers for the prediction of clinical response.
